# New data on David's myotis, *Myotis davidii* (Peters, 1869) (Mammalia, Chiroptera, Vespertilionidae), in Siberia and the Urals

**DOI:** 10.3897/BDJ.7.e34211

**Published:** 2019-04-25

**Authors:** Alexander Zhigalin

**Affiliations:** 1 National Research Tomsk State University, Tomsk, Russia National Research Tomsk State University Tomsk Russia

**Keywords:** Myotinae, *
Myotis
*, Siberia, Altai-Sayan, Ural, Distribution

## Abstract

**Background:**

David's myotis, *Myotis davidii*, is a vespertilionid bat inhabiting the wide spaces of the Palearctic region. Although previously registered in the north of Mongolia (50° N.L.) and the southern Urals (52° N.L.), data on the ecology of the species on the northern periphery of the range was missing.

**New information:**

The northern border area of *M. davidii* in Siberia shifts by 350 km and the area increases by about 150,000 km^2^, in the Ural area by 150 km. Pups in the north of the range appear from the second half of June to July inclusive. Our data reveal that individuals from the Urals and the North Caucasus are genetically similar.

## Introduction

Although David's myotis, *Myotis davidii* (Peters, 1869), was previously considered a subspecies of the common whiskered myotis, *Myotis mystacinus* (Kuhl, 1817), molecular evidence and detailed analyses of the skull and teeth morphology, however, confirmed its distinction (Benda and Tsytsulina 2000, Tsytsulina et al. 2012). *Myotis aurascens* Kuzyakin, 1935 was previously used as the valid name for the species, which was replaced by *M. davidii* (see Benda et al. 2012). These changes in the nomenclature must be taken into account when analysing literature and searching for sequences in Genbank.

The range of *M. davidii* includes the south and southeast of Europe, the Caucasus, Small, Middle, Western and Central Asia, the Himalayas, Western and Northern China and Korea (Benda and Tsytsulina 2000, Benda et al. 2012, Tsytsulina et al. 2012). The northern boundary of the range within Siberia and the Urals is along the Transbaikal region, Mongun-Taiga mountain and Southern Urals.

According to the IUCN Red List, the territory of Central and Central Asia does not belong to the habitat of *M. davidii* (Benda and Paunović 2016, Smith et al. 2008). Currently, the boundaries of the range of *M. davidii* are not well defined and very little is known on its distribution and ecology (Benda et al. 2012, Tsytsulina et al. 2012, Snitko and Snitko 2018, Smith et al. 2008). In the absence of these data, conservation actions to protect the species in the northern border of its distribution range are not possible. In addition, the study of the species on the periphery of the range is extremely important for fundamental research. This is due to the fact that it is assumed that peripheral populations are distinguished by their genetic structure, characteristics of their distribution and adaptation mechanisms (Eckert et al. 2008). Information about peripheral populations is needed to solve the central-peripheral hypothesis (Garner et al. 2004, Hengeveld and Haeck 1982).

The purpose of this study was to identify the northern limit of the distribution of *M. davidii* and some aspects of its ecology.

## Materials and methods

### Study Region

The Altai-Sayan region and Southern Urals are hotspots of biodiversity. These regions are amongst the priority areas for protection in international programmes such as Global 200 (Olson and Dinerstein 2002), Frontier Forests (Bryant et al. 1997) and Last of the Wild (Sanderson et al. 2002). A biodiversity study of these regions is necessary to monitor the state of ecosystems and develop conservation measures. At the same time, these territories are extremely inaccessible for researchers and, therefore, their biodiversity is poorly understood.

The Altai-Sayan regions contain and share their name with the Altai Mountains and the Sayan Mountains. The Altai Mountains constitute a mountain range in East-Central Asia, where Russia, China, Mongolia and Kazakhstan come together and are where the rivers Irtysh and Ob have their headwaters. The Sayan Mountains lie between north-western Mongolia and southern Siberia. The Altai-Sayan comprises 1,065,000 square kilometres. The region has high biodiversity, as it is located in transition zones between different ecoregions, altitudes and climate zones. It is in the Palearctic ecozone, with a Cold semi-arid climate. Mountain areas are covered with taiga forests, in intermountain basins steppe and semi-desert vegetation. Mountainous areas are covered with forests, and in the foothill areas – forest-steppe.

Southern Ural: the south, the widest part of the Ural Mountains, stretches from the river Ufa (near the village of Lower Ufaley) to the Ural River. From the west and east of the Southern Ural, the area is limited to the East European, West Siberian Plain and the steppes near the Aral Sea and Caspian Sea. The length of the Southern Ural is 550 km. The relief of the Southern Ural is more complex, with numerous valleys and parallel ridges directed south-west and meridionally. The climate of the Urals is continental. The average January temperatures are −15°C (5°F) in July are 20°C (68°F). The eastern areas receive 300–400 mm (12–16 in) precipitation. Maximum precipitation occurs in the summer. The winter is dry because of the Siberian High.

Fieldwork was conducted from 2012 to 2017 on the territory of the Uvs Lake Basin, Tuva Depression, Tannu-Ola mountains, Sayan Mountains and Uraltau range.

### Methods

We captured bats using mist-nets (3 x 5 m, 14 mm mesh; Mitchell-Jones and McLeish 2004). In the captured individuals, the species, sex and age were determined; and the length of the forearm, body, tail, thumb, tibia, hind foot and ear were measured (Mitchell-Jones and McLeish 2004). After inspection, all animals were released.

Species identification was performed according to the following morphological features (Dietz and Helversen 2004, Tsytsulina et al. 2012): length of the forearm < 36 mm, large dimensions of the thumb (5.2–7.0 mm, usually > 5.4 mm), lower leg (15.7–18.1 mm, usually > 16.1 mm) and hindfoot (6.8–8.7 mm, usually > 7.0 mm), forearm-length: 32.0–37.4 mm, length of fifth finger: 43 – 50 mm, length of third finger: 52 – 61 mm. The hind foot length is less than half of the tibia length. The wing membrane is inserted at the base of the outer toe. Spur length is no more than half the length of the margin of the tail membrane and there are no terminal lobes or breaks present. Posterior margin of the ear has a distinct indentation. Hair with dark bases and lighter tips, frequently with golden gloss. Upper second premolar rather small (maximum 1/2 of the size of the first upper premolar) and sometimes displaced palatally of the tooth row. Singular cusp of third upper premolar is small or absent, always lower than the second upper premolar. Ears brown, the inside of the ear and the base of the tragus lighter brown, sometimes even pinkish. Nostril often heart-shaped, lateral part usually well developed. Adult individuals always without yellowish-brown hair on the sides of the neck, therefore ventral and dorsal colours of the fur sharply divided.

Identification of a number of individuals was confirmed using molecular genetic methods. Tissue samples were collected from individuals in the field using a 4 mm diameter Keyes cutaneous punch (Surgical Access Pty Ltd.), as per Worthington-Wilmer and Barratt (Worthington-Wilmer and Barratt 1996) and the tissue stored in 95% ethanol. DNA isolation was performed according to the protocol attached to the DNeasy Blood Tissue Kit (250) of QIAGEN. Primers L2985 (5'-CCT CGA TGT TGG ATC AGG-3') and H4419 (5'-GTA TGG GCC CGA TAG CTT-3') by Ruedi and Mayer (2001) were selected for PCR mitochondrial ND1 gene. Amplification was carried out using the programme, which involved 94°С (3 min), 47 cycles at 94°С (45 s), 50°С (45 s), 72°С (47 s); 72°C (5 min). Sample sequencing was performed by Sintol (Moscow). Genetic sequences were deposited in GenBank: MK292722, MK321325, MK321326.

Sequence alignment was performed by BioEdit 7.0.5.3. The evolutionary history was inferred by the Tamura-Nei model. It was possible to obtain a correlation of the corrective approach and the corrective approach. The number of substitutions per site has been measured. This analysis involved 11 nucleotide sequences. There were a total of 730 positions in the final dataset. Evolutionary analyses were conducted in MEGA X (Kumar et al. 2018). For comparison, sequences from GenBank were taken: *M. aurascens* (AY699856 – AY699861), *Vespertilio murinus* L., 1758 (AY033964), *Miniopterus schreibersii* Kuhl, 1817 (AY033969) (Tsytsulina et al. 2012).

The Ethics Committee of the National Research Tomsk State University has approved procedures for catching and inspection bats and taking samples for genetic studies (permit number: 02.04.2011; 13.06.2013; 06.04.2015).

## Taxon treatments

### Myotis davidii

(Peters, 1869)

#### Materials

- **Type status:** Other material. **Occurrence:** individualCount: 1; sex: female; lifeStage: adult; **Taxon:** scientificName: Myotis davidii; kingdom: Animalia; class: Mammalia; order: Chiroptera; family: Vespertilionidae; genus: Myotis; specificEpithet: davidii; taxonRank: species; scientificNameAuthorship: Peters, 1869; **Location:** continent: Asia; country: Russia; stateProvince: Tyva Republic; verbatimCoordinates: 50°65'27"N, 93°74'31"E; **Event:** eventDate: 07/15/2016; habitat: Gallery forest
- **Type status:** Other material. **Occurrence:** individualCount: 2; sex: female; lifeStage: adult; **Taxon:** scientificName: Myotis davidii; kingdom: Animalia; class: Mammalia; order: Chiroptera; family: Vespertilionidae; genus: Myotis; specificEpithet: davidii; taxonRank: species; scientificNameAuthorship: Peters, 1870; **Location:** continent: Asia; country: Russia; stateProvince: Tyva Republic; verbatimCoordinates: 50°67'80"N, 93°00'73"E; **Event:** eventDate: 07/17/2016; habitat: lambing barn
- **Type status:** Other material. **Occurrence:** individualCount: 1; sex: male; lifeStage: adult; **Taxon:** scientificName: Myotis davidii; kingdom: Animalia; class: Mammalia; order: Chiroptera; family: Vespertilionidae; genus: Myotis; specificEpithet: davidii; taxonRank: species; scientificNameAuthorship: Peters, 1871; **Location:** continent: Asia; country: Russia; stateProvince: Tyva Republic; verbatimCoordinates: 50°65'34"N, 94°40'44"E; **Event:** eventDate: 07/18/2017; habitat: lambing barn
- **Type status:** Other material. **Occurrence:** individualCount: 14; sex: male and female; lifeStage: adult and young; **Taxon:** scientificName: Myotis davidii; kingdom: Animalia; class: Mammalia; order: Chiroptera; family: Vespertilionidae; genus: Myotis; specificEpithet: davidii; taxonRank: species; scientificNameAuthorship: Peters, 1872; **Location:** continent: Asia; country: Russia; stateProvince: Tyva Republic; verbatimCoordinates: 50°50'79"N, 94°74'49"E; **Event:** eventDate: 07/23/2017; habitat: road bridge
- **Type status:** Other material. **Occurrence:** individualCount: 1; sex: female; lifeStage: adult; **Taxon:** scientificName: Myotis davidii; kingdom: Animalia; class: Mammalia; order: Chiroptera; family: Vespertilionidae; genus: Myotis; specificEpithet: davidii; taxonRank: species; scientificNameAuthorship: Peters, 1873; **Location:** continent: Asia; country: Russia; stateProvince: Tyva Republic; verbatimCoordinates: 50°24'12"N, 94°76'03"E; **Event:** eventDate: 07/24/2017; habitat: Gallery forest
- **Type status:** Other material. **Occurrence:** individualCount: 1; sex: female; lifeStage: adult; **Taxon:** scientificName: Myotis davidii; kingdom: Animalia; class: Mammalia; order: Chiroptera; family: Vespertilionidae; genus: Myotis; specificEpithet: davidii; taxonRank: species; scientificNameAuthorship: Peters, 1874; **Location:** continent: Asia; country: Russia; stateProvince: Tyva Republic; verbatimCoordinates: 52°02'45"N, 94°41'01"E; **Event:** eventDate: 07/25/2017; habitat: Gallery forest
- **Type status:** Other material. **Occurrence:** individualCount: 4; sex: male and female; lifeStage: adult; **Taxon:** scientificName: Myotis davidii; kingdom: Animalia; class: Mammalia; order: Chiroptera; family: Vespertilionidae; genus: Myotis; specificEpithet: davidii; taxonRank: species; scientificNameAuthorship: Peters, 1875; **Location:** continent: Asia; country: Russia; stateProvince: Krasnoyarsk Krai; verbatimCoordinates: 51°80'23"N, 92°13'94"E; **Event:** eventDate: 07/13/2012; habitat: Gallery forest
- **Type status:** Other material. **Occurrence:** individualCount: 1; sex: female; lifeStage: adult; **Taxon:** scientificName: Myotis davidii; kingdom: Animalia; class: Mammalia; order: Chiroptera; family: Vespertilionidae; genus: Myotis; specificEpithet: davidii; taxonRank: species; scientificNameAuthorship: Peters, 1876; **Location:** continent: Asia; country: Russia; stateProvince: Krasnoyarsk Krai; verbatimCoordinates: 51°85'66"N, 92°16'14"E; **Event:** eventDate: 07/15/2012; habitat: Gallery forest
- **Type status:** Other material. **Occurrence:** individualCount: 2; sex: female; lifeStage: adult; **Taxon:** scientificName: Myotis davidii; kingdom: Animalia; class: Mammalia; order: Chiroptera; family: Vespertilionidae; genus: Myotis; specificEpithet: davidii; taxonRank: species; scientificNameAuthorship: Peters, 1877; **Location:** continent: Asia; country: Russia; stateProvince: Krasnoyarsk Krai; verbatimCoordinates: 51°91'33"N, 91°97'60"E; **Event:** eventDate: 07/23/2012; habitat: Gallery forest
- **Type status:** Other material. **Occurrence:** individualCount: 2; sex: male; lifeStage: adult; **Taxon:** scientificName: Myotis davidii; kingdom: Animalia; class: Mammalia; order: Chiroptera; family: Vespertilionidae; genus: Myotis; specificEpithet: davidii; taxonRank: species; scientificNameAuthorship: Peters, 1878; **Location:** continent: Asia; country: Russia; stateProvince: Bashkortostan Republic; verbatimCoordinates: 53°28'45"N, 58°39'35"E; **Event:** eventDate: 08/19/2014; habitat: Gallery forest

#### Diagnosis

**Figure 1.**
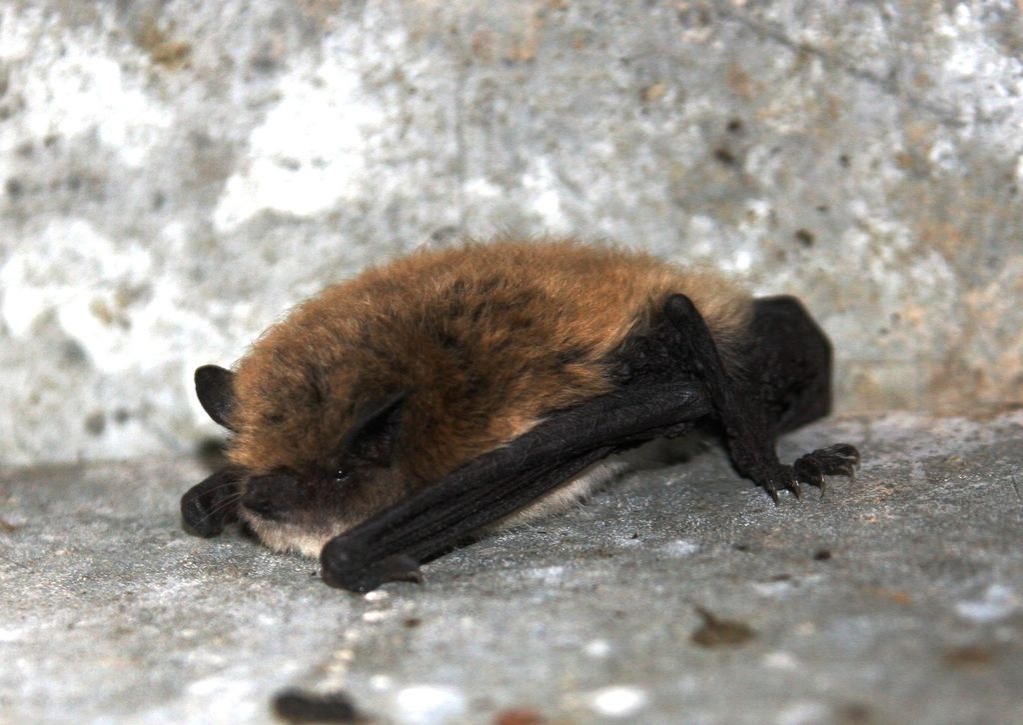
David's myotis from Yenisei river.

*M. davidii* is morphologically similar to *M. mystacinus*, *M. ikonnikovi*, *M. brandtii* and *M. sibiricus*. It differs from other species by the smaller forearm, lower leg; singular third upper premolar is small or absent, when present, it is always lower than the second upper premolar; paraconuli absent (Dietz and Helversen 2004, Tsytsulina et al. 2012). The base of the hair is dark, the tip is light, the wing membranes and ears are dark Fig. 1

#### Results

A total of 28 *M. davidii* individuals were caught: 26 in the Altai-Sayan region and 2 in the Southern Urals (Table 1). All animals were from the terrains located beyond the known limits of distribution of this species.

## Discussion

**Altai-Sayan region.** Prior to the present investigations, the extreme northeast locality of *M. davidii* in the Altai-Sayan region was the village of Mugur-Aksy (50°36'39''N, 90°65'15"E), Mongun-Tayginsky district Tuva Republic (EMBL 2019). Other previous records of the species in the region were made in Central and Southern Mongolia (Datzmann et al. 2012).

**Figure 2.**
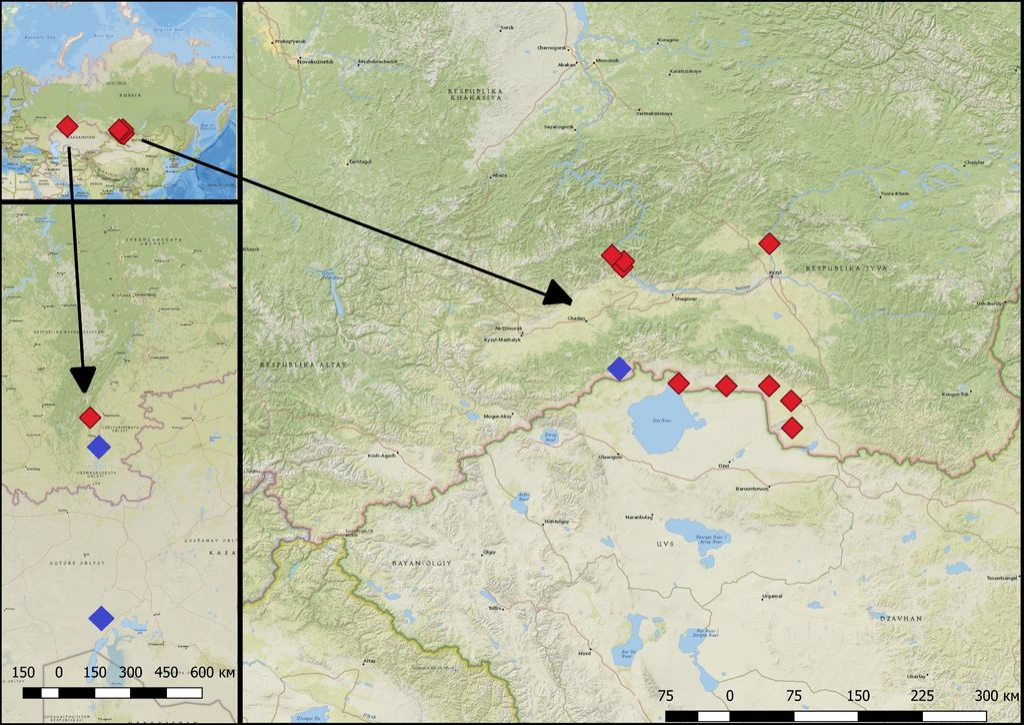
Previously known occurrence records (blue) and new occurrence localities (red) for *M. davidii* in the Uvs Lake Basin, Tuva Depression, Tannu-Ola mountains, Sayan Mountains and Urals.

We captured 26 individuals in the region in 9 localities (Fig. 2). New sites are located in Uvs Lake Basin, Tuva Depression, Tannu-Ola mountains and Sayan Mountains. These records push the known distribution boundary to the southern slopes of West Sayan . Thus, the northern border for its distribution range is shifted by 350 km and the area increased by about 150,000 km^2^.

**Figure 3.**
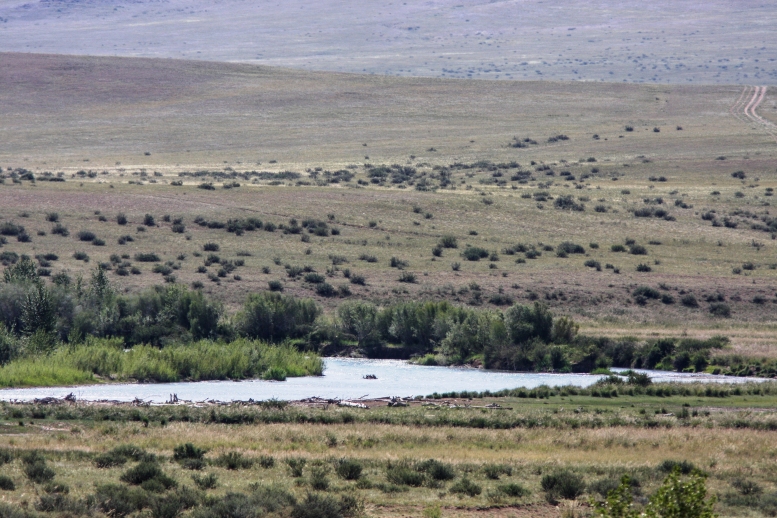
Tes river. Fourteen individuals of *M. davidii* were captured with ground-level mist-nets.

**Figure 4.**
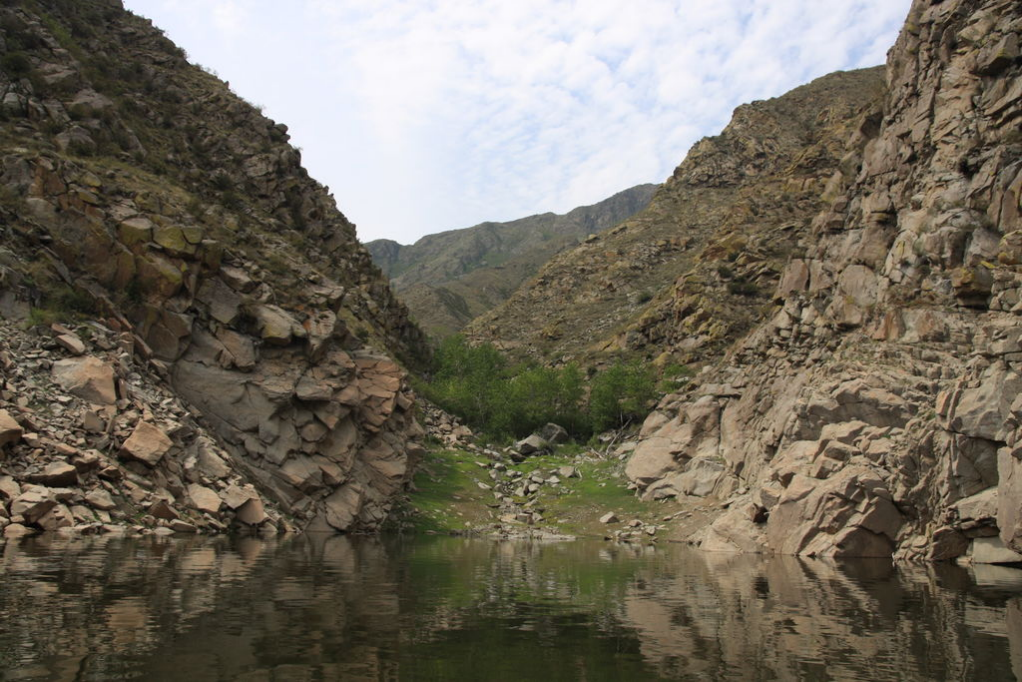
Gallery forests in West Sayan. One individual of *M. davidii* was captured with ground-level mist-nets.

**Figure 5.**
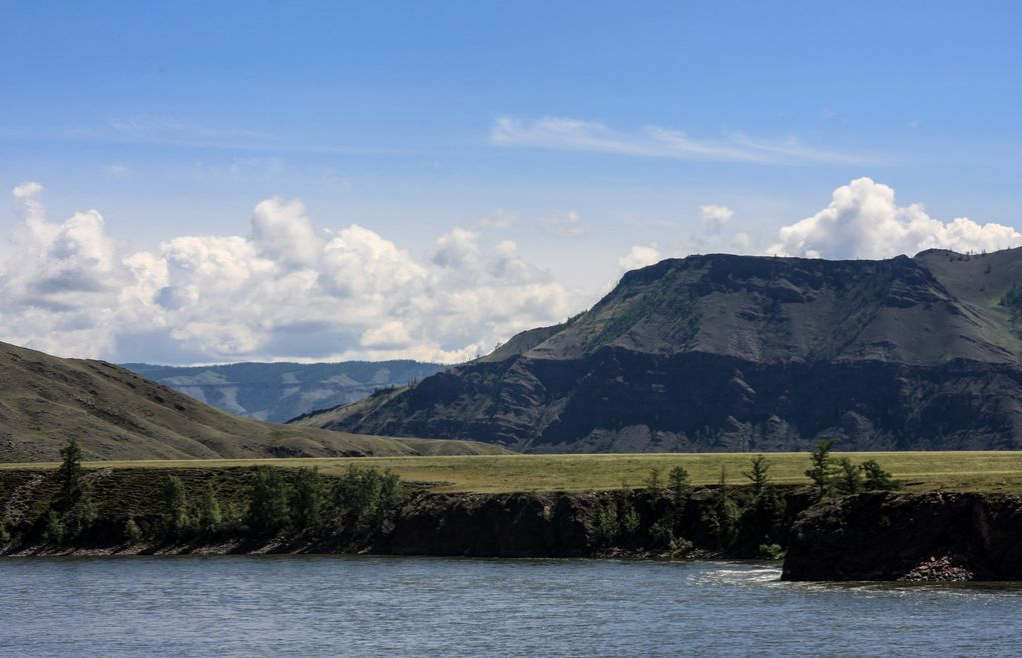
West Sayan, Yenisei river. Two individuals of *M. davidii* were captured with ground-level mist-nets.

**Figure 6.**
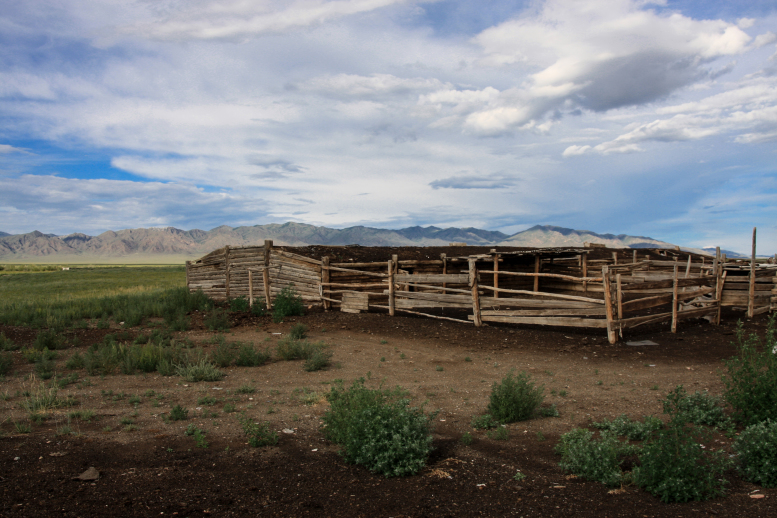
Lambing barn in Uvs Lake Basin. Two individuals of *M. davidii* were captured with ground-level mist-nets.

Most of the animals in the Altai-Sayan region were captured over the rivers near gallery forests (Figs 3, 4, 5), whereas 3 individuals were found during the day in single buildings (lambing barn; Fig. 6) аmongst the vast steppe spaces.

We found the first maternity colony in the region under the road bridge across the Tes river (4 localities,) on July 23. Nine adult females, 3 young females and 2 young males were trapped under the bridge. Amongst the fingerlings, 3 flew on their own, while 2 were newborn and attached to their mothers. These data indicate a prolonged period of young stock appearance, the onset of which is presumably in the middle of June.

**Ural.** The extreme northern locality of *M. davidii* in the Urals is near the settlement, Ural (52°61'55''N, 58°99'19''E) Chelyabinsk oblast (Snitko and Snitko 2018). Voucher specimens are deposited in the Zoological Museum Ilmen Nature Reserve (HC 70637–70639).

**Figure 7.**
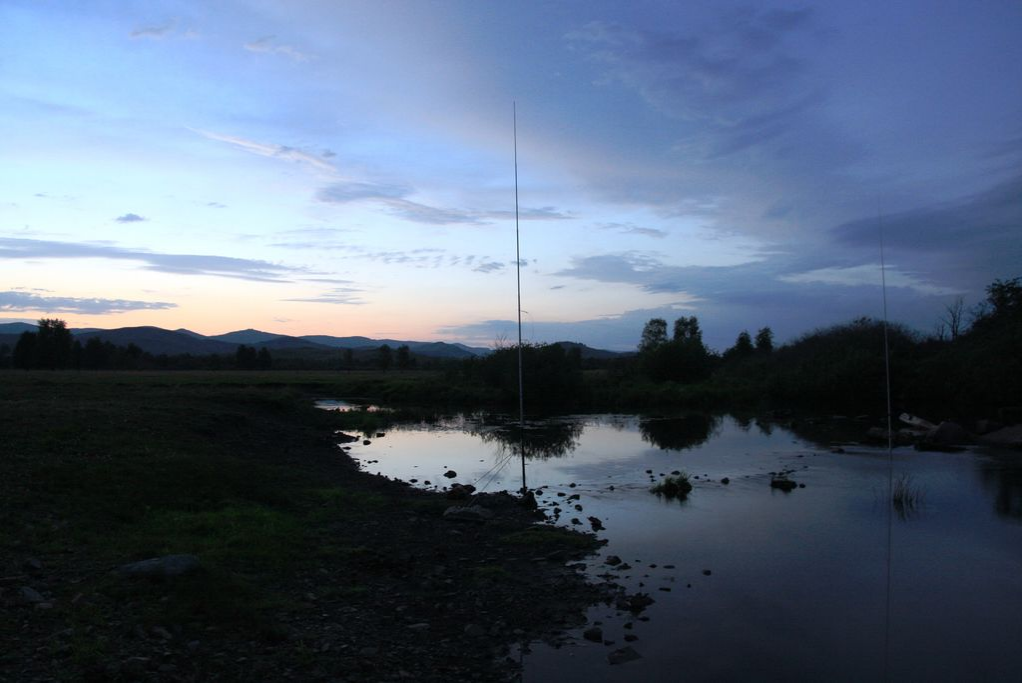
Bol'shoj Kizil river. Two individuals of *M. davidii* were captured with ground-level mist-nets.

We captured 2 adult males of *M. davidii* on the Bol'shoj Kizil river (Fig. 7), which is 150 km north of the previously known find. *Myotis mystacinus* were captured in the same locality. Thus, these cryptic species occur in sympatry.

**Figure 8.**
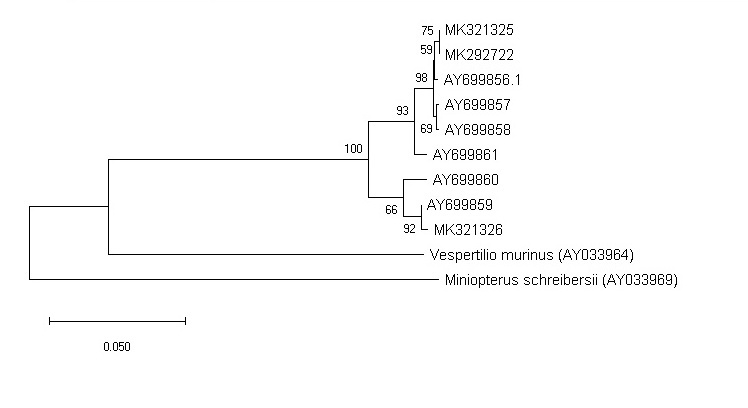
Maximum Likelihood tree (Tamura-Nei model).

Previously, studies have been conducted on the genetic structure of the species (Tsytsulina et al. 2012). Our analysis (Fig. 8) showed that *M. davidii* from the Southern Urals (MK292722, MK321325) is closest to that of the North Caucasus (AY699856). An individual from Tuva (MK321325) is closest to the animal AY699859.

In general, our studies have shown that the northern boundary of the distribution range of *M. davidii* is 150-350 km to the north. In Southern Siberia, the species occurs not only in mountainous areas, but also in intermontane basins, where it is usual for them to settle in gallery forests and human buildings. The breeding period is stretched and falls in June-July. Our results have shown that animals from the Southern Urals and the Caucasus form a common clade.

Due to the species occurrence in the administrative territories of Russia (Krasnoyarsk Territory, Tuva, Bashkiria), where *M. davidii* was not previously reported and its rarity on the northern periphery of the range, the conservation status of *M. davidii* should be locally addressed.

## Supplementary Material

XML Treatment for Myotis davidii

## Figures and Tables

**Table 1.** Information on the sampling sites. Geographical coordinates are given in the format of decimal degrees (DD).

№	Location	Exact location data (Lat. / Lon.)	Number of animals (ad - adult; juv - juvenis)
1	*Altai*-*Sayan region*, Uvs Lake Basin, Despen	50°65'27''N, 93°74'31''E	1♀ ad
2	*Altai*-*Sayan region*, Uvs Lake Basin	50°67'80''N, 93°00'73''E	2♀♀ ad
3	*Altai*-*Sayan region*, Uvs Lake Basin	50°65'34''N, 94°40'44''E	1♂ad
4	*Altai*-*Sayan region*, Uvs Lake Basin Tes river	50°50'79''N, 94°74'49''E	9 ♀♀ ad, 3 ♀♀ juv, 2 ♂♂ juv
5	*Altai*-*Sayan region*, Uvs Lake Basin	50°24'12''N, 94°76'03''E	1♀ ad
6	*Altai*-*Sayan region*, West Sayan, Bol'shoy Yenisei river	52°02'45''N, 94°41'01''E	1♀ ad
7	*Altai*-*Sayan region*, West Sayan, Yenisei river	51°80'23''N, 92°13'94''E	3♀♀ ad, 1♂ad
8	*Altai*-*Sayan region*, West Sayan, Yenisei river	51°85'66''N, 92°16'14''E	1♀ ad
9	*Altai*-*Sayan region*, West Sayan, Yenisei river	51°91'33''N, 91°97'60''E	2♀♀ ad
10	*Southern Urals*, Bol'shoj Kizil river	53°28'45''N, 58°39'35''E	2♂♂ ad
